# High-fidelity crop modelling: innovations driving sustainable fruit horticulture

**DOI:** 10.3389/fpls.2026.1902281

**Published:** 2026-08-06

**Authors:** Utpal Das, Annamalai Subbiah Mailappa, Silvia Nicolae, Shiva Sai Prasad, Madhusmita Dishri

**Affiliations:** 1 Vellore Institute of Technology School of Agricultural Innovations and Advanced Learning, Vellore Institute of Technology, Vellore, Tamil Nadu, India; 2 Department of Natural Resource Management, College of Horticulture and Forestry, Pasighat, Arunachal Pradesh, Central Agricultural University, Imphal, Manipur, India; 3 Research Institute for Fruit Growing, Pitești, Romania; 4 Department of Agriculture, Koneru Lakshmaiah Education Foundation, Vaddeswaram, Andhra Pradesh, India

**Keywords:** climate-smart horticulture, crop management, crop modelling, fruit crops, GIS, phenology modelling, precision agriculture, remote sensing

## Abstract

Effective decision support systems rely on a clear understanding of real-world conditions and on developing strategies to manage them. In the current era of severe climate change, crop modelling has emerged as a valuable tool in fruit production. It enables farmers and researchers to mitigate the adverse effects of biotic and abiotic stresses by providing scientifically grounded, practical, and forward-looking solutions. This review aims to evaluate the influence of climate and management practices on fruit quality and to highlight how crop modelling can address challenges related to crop growth and development. It focuses on enabling timely, informed decision-making to improve outcomes. These models help in understanding perennial fruit crops with respect to canopy architecture, plant structure, root and aerial systems, light interception, photosynthesis, respiration, carbon allocation, nutrient uptake, phenology, and pest and disease forecasting. Each model’s applications and limitations are considered to provide a comprehensive perspective. They facilitate the interpretation of experimental data and enhance understanding of complex biological systems. Dynamic and quantitative modelling approaches support timely decision-making, improve analysis of agricultural systems, and promote interdisciplinary research, ultimately increasing management efficiency. These technologies enhance decision-making across pre-harvest, harvest, and post-harvest stages, contributing to yield improvement and the wider adoption of precision agriculture, especially in developing countries.

## Highlights

- Highlights of previously used techniques for crop modelling
- Discussing functional−structural plant models (FSPMS) enhancing 3d canopy & fruit development simulation
- Recent techniques to develop functional−structural models in fruit crop modelling
- Predictive modelling in fruit crops

## 1 Introduction

Horticulture is a multifaceted discipline that demands strategic precision across the entire value chain. It requires a balance of technical expertise, such as varietal selection, phenological mapping, and pest management at both micro- and macro-levels, with operational efficiency in resource optimisation and the judicious use of crop protectants. Beyond the field, success hinges on accurate yield forecasting, sophisticated post-harvest handling, and a keen ability to navigate consumer trends and market dynamics. Fruit trees are perennials in nature and require complex modelling for nutrient uptake, photosynthesis, canopy architecture, pest forecasting, etc. Currently, the escalating impact of climate change is inflicting severe losses on existing germplasm and farmer revenue, and the emergence of unprecedented pests and diseases. Defined as the “schematic representation of the system” model (de Wit et al., 1970). In this context, implementing crop modelling will be instrumental in protecting crops from the detrimental effects of biotic and abiotic stresses.

In recent years, crop growth models have been effectively implemented globally across a variety of pedoclimatic, genotypic, and management conditions (Gary et al., 1998). Their application is a significant response to the numerous challenges associated with understanding the intricate nature of the soil-plant-atmospheric continuum and the increasing demand for innovative approaches to address emerging technological advancements and datasets, and to enhance crop management strategies, thereby addressing food security needs. The horticulture community has access to an extensive database regarding crop modelling. The International Society for Horticultural Science (ISHS) has made a significant contribution to this field through the “Modelling plant growth, environmental control and greenhouse environment” group, the “computer modelling in fruit research and orchard management” group, and the “Timing field production of vegetables” group. However, implementing these highly technical systems comes with several practical, biological, and computational challenges, such as high data demands and biological complexity with mathematical simplification (Silva and Giller, 2020). The simulation models for annual field crops have come a long way, but creating reliable models for perennial fruit trees remains difficult. This lag is largely due to the unique structural complexity of trees, their perennial lifecycles, and the intricate ways they respond to environmental changes and farming practices. To overcome such issues, many new technologies have emerged recently, some of which have proven very fruitful for policymakers when making decisions about food production. These decisions also carry the risk of an unknown population rise, an undetermined decrease in cultivable land, and, most importantly, unpredictable natural calamities that never knock before their arrival. In such situations, crop modelling helps overcome the problems associated with significant decisions (Thornley and Johnson, 1990).

A model is described as “an attempt to characterise a specific process or system via the use of a simplified representation, preferably a quantitative mathematical expression that concentrates on relatively few essential factors that affect the process or system”. In fact, a crop model is a well-conceived plan of action for growing the intended crop to produce the maximum possible yields with high nutrient levels under available resources. Modelling is used in combination with equations to explain the system’s behaviour. Crop models are computerised software-based tools that simulate crop growth and development of a particular plant. While Continuous developments in the field of crop models are crucial, there should be balance between level of complexity and the processes modelled, for the successful application of the models.

Crop models are representations of real systems and use mathematical techniques to simulate how a crop grows in response to its environment. It uses crop, soil, and weather data to predict crop growth and yield. These are simplified versions of the actual field situation in computerised form. The crop models include all the factors of a basic crop system and, in some cases, additional factors for specific crops. As agricultural systems are quite complex and difficult to understand, modelling helps simplify them, but it also introduces drawbacks for accurate results. Experience from past research has shaped the focus and application of crop models in response to societal needs (Dorey et al., 2015). They have contributed to decision support (Gutierrez-Galan et al., 2018) and risk assessment (Heuvelink, 1999).

Modelling simulations hold the key to functions such as crop modelling, which helps mitigate climatic risks with specific implications. To replicate crop growth and development, computer programmes called “crop models” were developed (Hopf et al., 2022). Crop modelling helps reduce the requirements of a basic field experiment by operating it virtually in seconds without wasting time and money. Crop growth simulation models forecast yield and provide quantitative data on key stages in crop development. Crop models predict likely yields by accounting for production practices and inputs. This will ultimately help fulfil agricultural demands (Kumari et al., 2022). In the present era, fruit quality has become a crucial factor in fruit production. Thus, the emergence of this new era of modelling, especially functional-structural plant modelling, plays a pivotal role (Léchaudel et al., 2005). Recent research works are therefore reoriented with a focused aim of understanding the climate and management aspects and their impact on fruit quality, and, to achieve this goal, mathematical models serve as useful frameworks (Lescourret et al., 1998).

While prior literature has predominantly centred on annual crop simulations, this review bridges that gap by synthesising traditional crop models, functional-structural plant models, disease prediction tools, and modern machine learning techniques tailored specifically to fruit crops. Recent breakthroughs in AI, remote sensing, precision horticulture, and FSPM have dramatically enhanced the power of crop simulations. Consequently, there is a timely need for a fresh, comprehensive review centred exclusively on fruit-bearing horticultural systems. So, this review aims to critically assess the spectrum of crop modelling techniques currently employed in fruit cultivation, ranging from mechanistic, empirical, and simulation-based models to functional-structural and machine learning approaches. Additionally, it seeks to pinpoint existing constraints, examine emerging technological innovations, and delineate future research pathways to enhance the sustainability of fruit production amidst climate change.

## 2 A brief history of crop modelling

Crop modelling began 100 years ago, when, from 1920 to 1950, the first mathematical descriptions were integrated into models of plant and crop growth and soil processes (Keating and Thorburn, 2018). Modelling was first tried on sugar beet. The main reason for considering sugar beet is its simple morphology, consisting of two basic parts: the edible root and the leafy crown. Cereals were the next to be modelled. Gradually, it was applied to more complex systems, such as fruit crops. Among the various plant processes, the rate of photosynthesis in canopies was first attempted and modelled. Later, various models came into view, such as the crop growth simulator ‘ELCROS’(Elementary Crop Growth Simulator) (Miranda et al., 2008), the basic crop growth simulator ‘BACROS’ (Mokria et al., 2022), the crop environment resource synthesis (CERES) (Jones et al., 2003), A resource capture model (RESCAP) (Motisi et al., 2008), and agricultural land management alternative with numerical assessment criteria (ALMANACK) (Neamtu et al., 2008). However, some models are crop-specific and used for a specific purpose. For example, POTATO is used for potato crops, and COTTON is specifically used for the growth, development, soil water budget, and morphology of cotton crops. Currently, one of the most important models successfully used by many researchers is the system for decision support for agro-technology transfer (DSSAT), developed as a part of the international benchmark sites network by scientists with International Benchmark Sites Network for Agrotechnology Transfer project (IBSNAT) (Salazar et al., 2008; Pereira et al., 2017) model consists of a range of application packages that cover a huge spectrum of utility for various researchers.

## 3 Factors affecting the crop models

As agricultural models mimic real systems, all the factors influencing them must be considered in a crop model (Soltani et al., 1995; Salinas et al., 2019). Generally, four major factors are required as inputs in the model: climate, soil, crop, and management. Climate inputs such as the highest and lowest temperatures, rainfall, relative humidity, and daylight hours (Tixier et al., 2004; Sousa et al., 2022) affect crop growth and thus crop modelling. However, different models have different requirements for climatic input information based on their utility. The soil input incorporates all the information regarding the soil status of the experimental area (Tixier et al., 2004). Crop input includes all details of the crop used in the experiment, such as variety, duration, flowering, etc. The management factor is important as the practises involved in the experiment can greatly change the yield (Sousa et al., 2022). Crop genetic coefficients, which vary by crop and species, also play a significant role in modelling. Accurate calculation of this factor is important for a valuable outcome. However, some models have in-built genetic coefficient calculators, which make this easier. Once all required data has been entered into the software, the model outputs can be obtained.

Further, they are also affected by spatial heterogeneity—assuming a whole field or regional ecosystem grows uniformly. Remote sensing via satellites (e.g., Sentinel, Landsat) and Unmanned Aerial Vehicles (UAVs/drones) provides continuous spatial data like Leaf Area Index (LAI) and Normalised Difference Vegetation Index (NDVI) (Sun et al., 2025). Similarly, IoT (of Things) sensors track hyper-local, real-time data from the ground. Networked arrays of wireless soil moisture, sap flow, temperature, and relative humidity sensors feed data directly into crop software, yielding multi-spectral satellite imagery yields highly complex datasets.

## 4 Methodological framework in crop modelling

Crop modelling has three basic steps: model development, which includes crop-based models developed by software engineers with extensive support and information from across all sectors of agriculture. These models are based on information gathered from several sources across thousands of field experiments. Years of research have enabled the model system to incorporate as much information as possible, helping it better mimic real situations. Second, model calibration by evaluating the model output against the exact observed data. Calibration refers to modifying the model to match the field experiment. To calibrate a model, first the experimental inputs are entered into the software, and later it compares the model-generated results with the farm results; if both are approximately equal, the model is successfully calibrated; otherwise, it is not. However, model calibration isn’t easy. To calibrate a model, data from many trials are required to obtain a perfect result. Last step, model validation: by checking whether the model results are valid in a farm situation or not. To validate a calibrated model, data from a crop system are to be entered into the software. Here, it is important to note that these data sets should not be the same as those used for calibration. Later, the experiment is carried out in the field using inputs similar to those fed into the software system. If the actual outcome is similar to the virtual one, the validation process can be considered successful.

**Figure 1:**
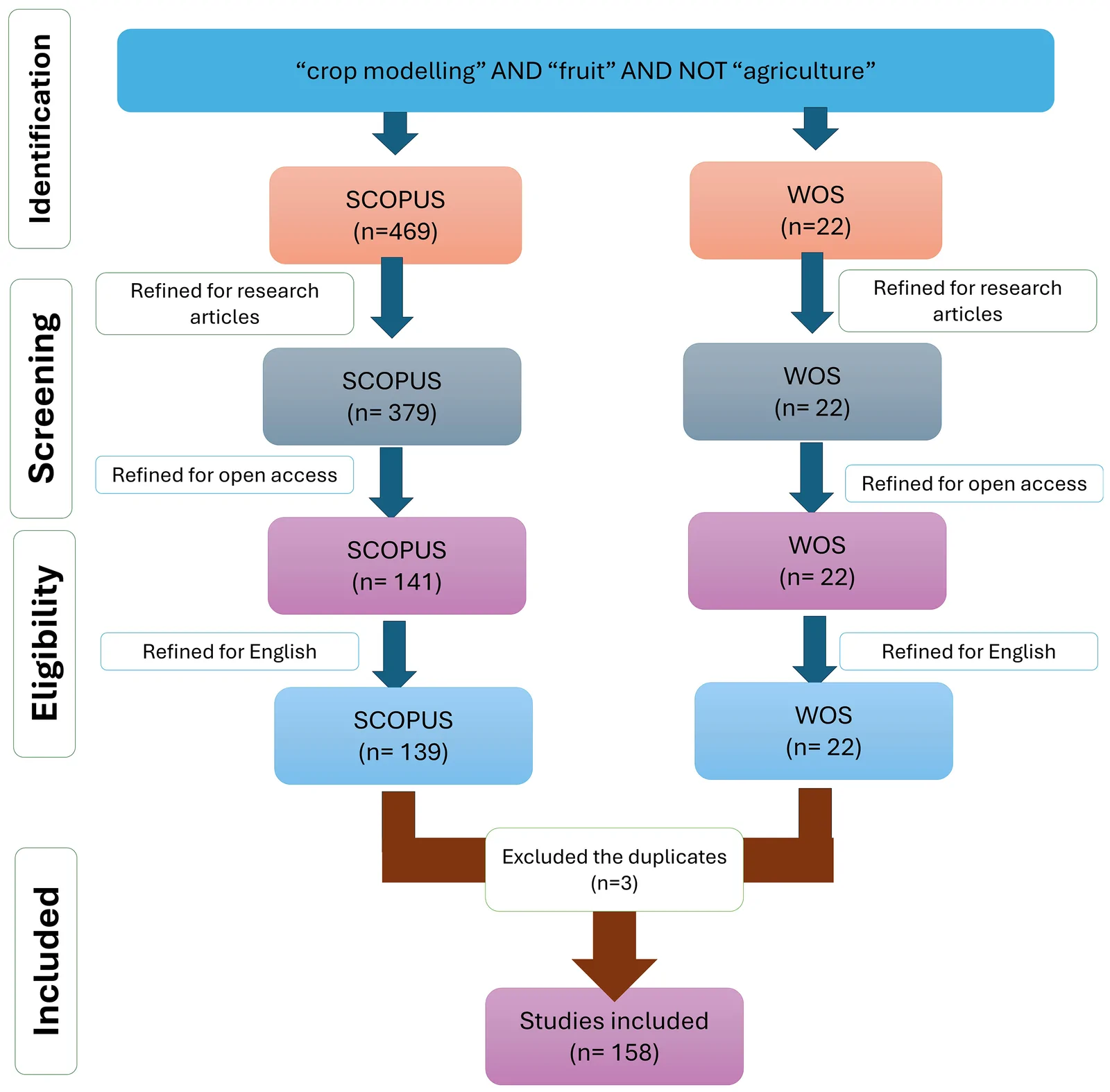
PRISMA technique followed for meta-analysis.

The research articles for the review have been selected using the PRISMA (Preferred Reporting Items for Systematic Reviews and Meta-Analyses) technique. The PRISMA technique is a fundamental framework that ensures transparency, structural integrity, and reproducibility in evidence-based research. When executing a meta-analysis—which statistically combines data from multiple independent studies—adhering to PRISMA’s 27-item checklist and standard 4-phase flow diagram is critical for establishing credibility (**Figure 1**). It systematically guides researchers through the explicit tracking of literature screening, from initial database discovery to the final selection of included trials from SCOPUS and WOS (Web of Science). The process includes the identification phase, in which identical search strings (“crop modelling” AND “fruit” AND NOT “agriculture”) are executed across both engines to capture initial record pools (SCOPUS = 469; WOS = 22). Further, non-original literature—such as book chapters, conference reviews, and editorial notes- was filtered out to isolate only peer-reviewed research articles (SCOPUS = 379; WOS = 22). Concurrently, filters are narrowed to open-access articles (SCOPUS = 141; WOS = 22), maximising the study’s transparency, public reach, and citation impact, and language filters are applied to retain only English articles to ensure uniform data extraction (SCOPUS = 139; WOS = 22). Because Scopus and WOS index overlapping journals, the next step is to consolidate the results and systematically remove duplicate records using the reference management software Zotero (n=3). Following this deduplication, the remaining records are subjected to predefined automated and manual filtering criteria to refine the study scope (n=158).

**Figure 2:**
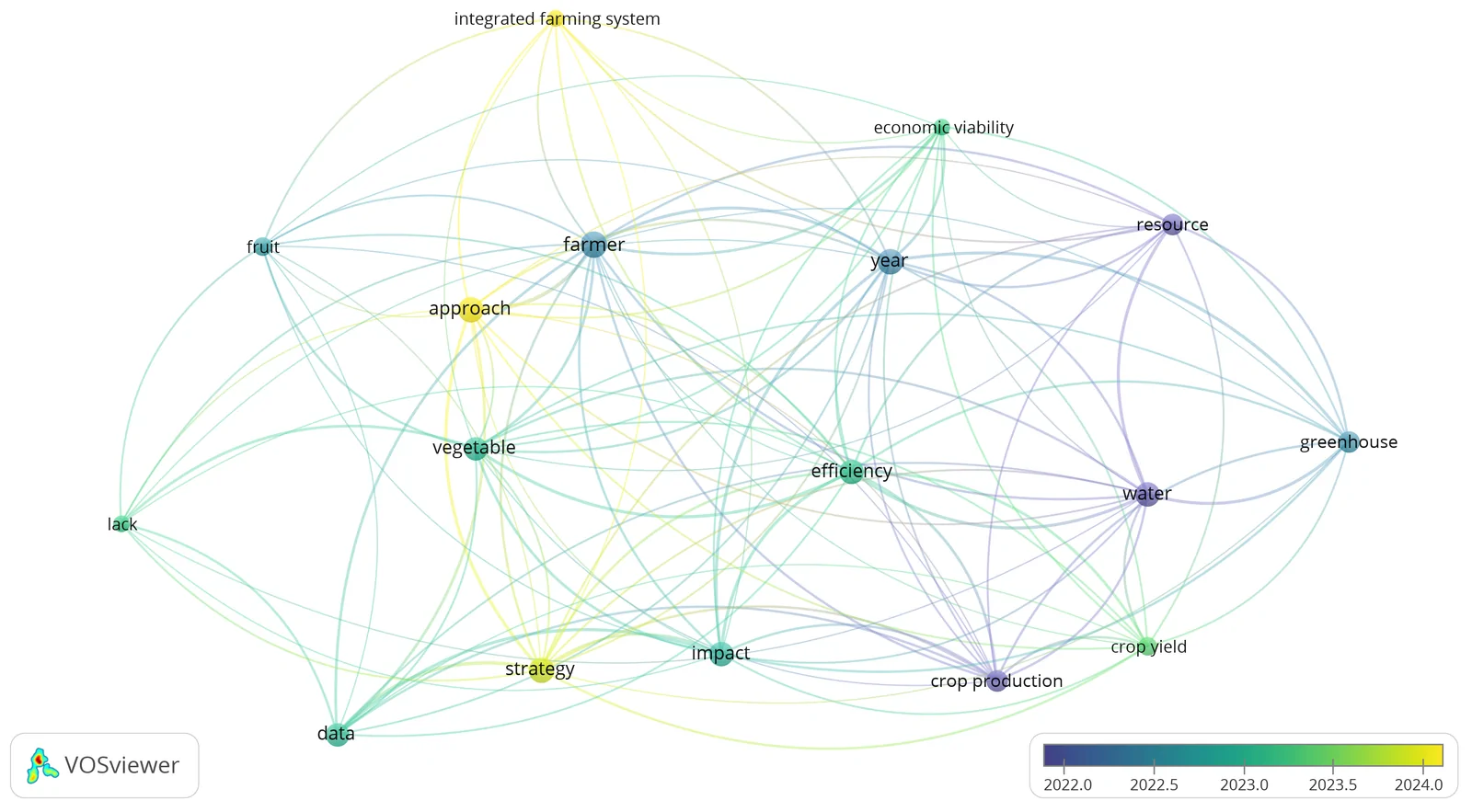
Network of keywords derived from 22 WOS articles related to crop modelling and fruit.

**Figure 3:**
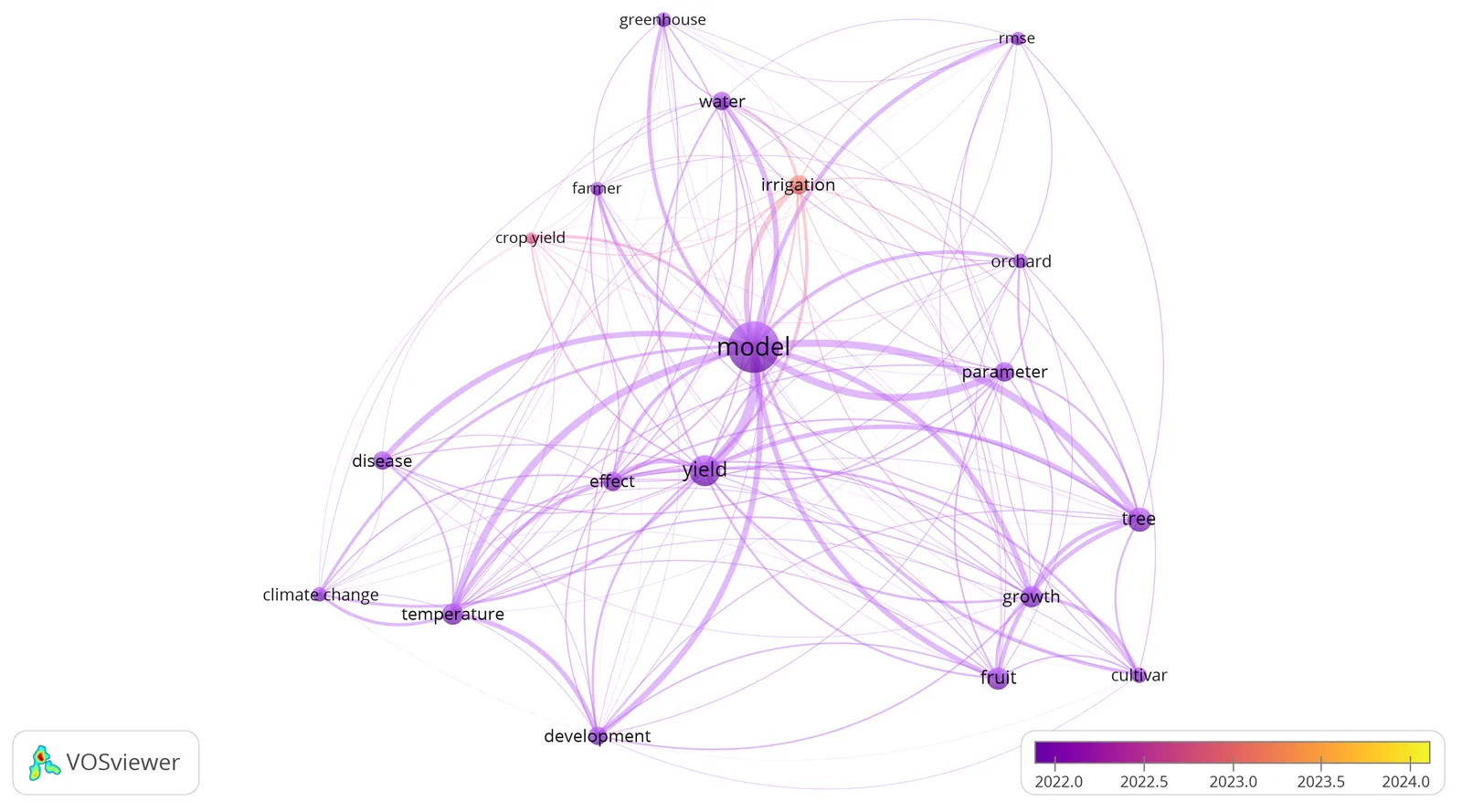
Network of keywords derived from 139 SCOPUS articles related to crop modelling and fruit.

Further, the data was analysed with VOSviewer software to understand the research conducted and published. By analysing keyword co-occurrences, the software automatically groups related terms into distinct, colour-coded thematic clusters, instantly revealing the sub-disciplines, core frameworks, and hidden research fronts of a scientific domain. When viewed through a temporal overlay, this network highlights how vocabulary evolves over time, shifting from foundational legacy topics to emerging cutting-edge trends. Ultimately, by visualising the strength of links and spatial distances between terms, it can pinpoint interdisciplinary bridges, expose structural research gaps, and clean up redundant synonyms—transforming raw bibliographic data into a strategic, scannable map of a field’s entire intellectual structure. The articles downloaded from SCOPUS and WOS were analysed separately using specific keywords for crop modelling. The WOS database was found to have 20 unique keywords for the query of each keyword repeating at least 15 times (**Figure 2**). Similarly, SCOPUS database was found to have 17 unique keywords for the query of each keyword repeating at least 5 times (**Figure 3**).

The details pertaining to the current practices in model calibration, validation, uncertainty quantification, and performance evaluation, in terms of performance evaluation metrics, sensitive analysis, uncertainly analysis and cross-validation (Saldaña Villota and Cotes Torres, 2021), are given below:

### 4.1 Performance metrics

Quantitative statistical metrics form the foundational basis for assessing model adequacy and prediction error.

Root Mean Squared Error (RMSE): RMSE measures the square root of the average squared errors between predicted and actual values. It penalises larger errors more heavily, making it sensitive to outliers.

Mean Absolute Error (MAE): MAE expresses prediction error as a percentage, facilitating comparisons across models and datasets. It is intuitive for communicating model accuracy in operational terms. It is more robust to extreme outliers than RMSE.

Coefficient of Determination (R²): R^2^ indicates the proportion of variance in the target variable explained by the model. Its popularity stems from its intuitive interpretation and widespread adoption in regression modelling. It quantifies the proportion of variance in the target variable that is explained by the model. It evaluates overall goodness-of-fit but does not penalise model complexity.

Nash-Sutcliffe Efficiency (NSE): Commonly used in environmental and hydrological modelling, NSE measures the relative magnitude of the residual variance to the measured data variance. It ranges from -∞ to 1, where an NSE of 1 signifies a perfect fit, and 0 indicates the model is no better than simply predicting the mean of the observed data.

### 4.2 Sensitivity analysis

Sensitivity analysis identifies which model parameters and input features exert the strongest influence on the predictions. This practice ensures that calibration efforts focus on the most impactful parameters, whilst also detecting non-identifiable or redundant parameters to prevent model overfitting.

### 4.3 Uncertainty analysis

Uncertainty analysis (often utilising frameworks like GLUE, SUFI-2, or fuzzy logic) quantifies the reliability of model outputs by evaluating input data errors and structural model limitations. It shifts the focus from a single deterministic prediction to producing confidence intervals, providing decision-makers with a probabilistic measure of risk.

### 4.4 Cross-validation

To guarantee that the model generalizes to independent, unseen data, cross-validation is essential. Techniques like K-fold cross-validation divide the dataset into multiple subsets, systematically utilising each subset for validation whilst the remainder is used for training. This mitigates dataset bias and ensures a robust evaluation of model parameters.

## 5 Understanding crop models categories

Models are classified into different categories: conceptual, physical, and mathematical (Acock and Acock, 1991). Conceptual models are hypothesis-centric and grounded in deep thought or imagination. In contrast, physical models are experimental in nature and not directly involved in explaining biological systems and subsystem units, representing a whole system. The third group, referred to as mathematical models, is the most extensively utilised group, in which the system’s behaviour is articulated through equations, allowing for the quantitative formulation of hypotheses based on deduced consequences. Among the mathematical models, several sub-classes exist, with empirical and mechanistic models regarded as the most essential (Acock and Acock, 1991). Both models can be deterministic, yielding precise quantitative predictions, or stochastic, encompassing a variety of distributions (González-Rodríguez et al., 2010).

### 5.1 Empirical models

Use of statistical relationships between inputs into the agricultural system (fertiliser, precipitation) and outputs (production), but cannot explain the fundamental processes (García-Tejera et al., 2024). These are simple regression equations (with one or more components) that are used to directly describe the observed data and estimate the final yield. Regression models describe the growth curve using empirical functions such as Richard’s function, polynomials, etc (Kumari et al., 2022).

### 5.2 Mechanistic models

This one justifies the system’s nature in terms of its more fundamental characteristics. Scientific principles were applied to describe crop growth and its interactions with the environment, using equations to simulate physiological processes within the plant (García-Tejera et al., 2024). These simulations can help us understand the physical, chemical, or biological mechanisms that underlie specific responses. In addition to the relationship between climatic parameters and yield, these models explain the underlying mechanism. These models are working, primarily based on physical selection (Vega et al., 2015). A mechanistic model dissects a system’s behaviour into its fundamental features, which are inherently mechanism-centric. These models can replicate essential physical, chemical, or biological processes and elucidate the mechanism of a certain linked reaction. These models elucidate the function of dependent variables in relation to their impact on a process (Oteng-Darko et al., 2013). The initial mechanistic model was process-based, concentrating on transpiration, photosynthesis, respiration, and their impact on plant growth. Plant growth is typically modelled as a process influenced by environmental conditions, including temperature, light, and CO_2_ concentration; some of these process-based models are presented in **Table 1**.

**Table 1:** List of some processed-based models, and their application on various fruit crops.

Name of the model	Species	Processes it related with	References
GREEN KIWIFRUIT	*Actinidia deliciosa*	Carbon Acquisition and Utilisation Hydrolysis Carbon Storage Restoration Perennial Biomass Maintenance	(Buwalda et al., 1990)
PEACH	*Prunus persica*	Photosynthesis Growth Respiration Carbon supply and demand Carbohydrate allocation	(DeJong and Grossman, 1995)
ALMOND	*Prunus dulcis*	Carbon partitioning, growth, photosynthesis, respiration, carbon supply and demand	(Rosati et al., 1999)
QualiTree	*Prunus persica*	Horticultural practices such as thinning, pruning and irrigation influence fruit quality and growth.	(Lescourret et al., 1998)
VitiSim	*Vitis vinifera*	Carbon allocation and carbon balance, organ respiration and daily photosynthesis rate	(Miras-Moreno et al., 2021)
OliveCan	*Olea europaea*	Water balance: uptake of water by roots, evaporation from soil, drainage and precipitation Carbon balance: partitioning of assimilates, maintenance, growth and respiration	(López-Bernal et al., 2023)
MaluSim	*Malus domestica*	Fixed carbon, respiratory costs, carbon exchange between plants and effects of environmental changes and agricultural practices on the dry matter.	(Lordan et al., 2019)
V-Mango	*Mangifera indica*	Carbon and water dynamics at the level of fruiting branches yield insights into fruit development.	(Rathod and Mishra, 2018)

### 5.3 Static and dynamic models

This model expresses the relationship between yield and weather parameters. In this type of model, relationships are measured using static techniques (correlations, top-down regressions) (Bharath et al., 2023). Even though the results of cropping systems are cumulative over time, a static model does not account for time as a variable. Based on historical datasets of crop and climate variables, crop-response models have recently been used to assess crop responses to climate change (Yu et al., 2019; Zhu et al., 2021).

### 5.4 Simulation model

These models collectively form a set intended to imitate a system’s behaviour. These models utilise one or more sets of differential equations to calculate both rate and state variables over time, from planting to the final harvest maturity. They take into account day-to-day variations in weather and soil conditions because they were designed to closely resemble the system over a minimal period (Oteng-Darko et al., 2013).

### 5.5 Stochastic model

Stochastic models yield various outputs based on the likelihood of each combination of fixed inputs. Developing a stochastic model that provides an expected mean value with corresponding variance, particularly when variability is considerable, is extremely desirable. It is recommended to employ these stochastic models whenever satisfactory results cannot be achieved through experimentation. The primary advantage of these models is that they provide multiple outputs, each with a probability associated with the input set (Torkashvand et al., 2017).

### 5.6 Deterministic model

Accurate predictions for quantities, without any probability distribution, variance, etc., are possible with the help of the deterministic models. Whenever the system is more uncertain, these deterministic models are less useful, for example, in rainfall prediction. These models calculate the precise yield value. A deterministic model presumes quantities such as crop yield or rainfall without incorporating volatility, probability distribution, or stochastic factors (Kumari et al., 2022).

### 5.7 Optimising models

Optimising models provide the optimal solution for a system’s effective functioning, encompassing a critical set of instructions that address the complex issue. These models are designed with the specific purpose of determining the appropriate management inputs for the system’s actual operation (Kumari et al., 2022).

## 6 Overview of existing crop models for fruit crops

In recent times, the quality of fruits has become an increasingly important aspect of production, leading to the emergence of a new field, termed functional-structural plant modelling (Léchaudel et al., 2005). Most of the models to date are mostly for field crops. Fruit crop models are very few, and this might be because of the complex physiology of the fruit crops. Most fruit trees possess branches, perennial trunks, and scaffold roots of several species, as well as floral buds’ location and behaviour during flowering, in addition to a huge number of vegetative and reproductive organs in the present year. As a result of its eternal existence, life relies on earlier years in various ways each year. Although they offer carbon and nutrient reserves, these permanent structures also have repercussions from year to year. It is difficult to create a multi-year model due to the complexity of carry-over effects on growth or cropping. Fruit trees can also be identified by their discontinuous canopy structures, strong management techniques such as pruning and training, and rootstock grafting, which provides a range of inconsistent effects. However, the effects on cropping and growth are little understood. For each fruit, different models have been proposed, and still, for some fruits, we didn’t have enough models either. So here we are discussing some fruit models that help improve the physiological and quality attributes of different fruits. We have different types of models, including statistical, linear, simulation, and mixed models. Each model has its own specifications, and each helps improve fruit quality and yield (Léchaudel et al., 2005).

### 6.1 FSPM

These models can be used to model the dimensional architecture of the plant as a function of physiological processes and environmental factors (Thornley and Johnson, 1990). These models combine structural information with physiological processes, making them useful tools for describing crop growth and development. Different modelling strategies have been used across several models with different elementary units to achieve this efficiently (White, 1984; Room et al., 1994). The working principle of these models is that “Metamer/phytomer” is the basic unit, consisting of a node with an axillary leaf, axillary buds, and an internode (White, 1984). “Growth unit,” a segment of an axis resulting from continuous elongation (Hallé et al., 1978), “Axis,” defined as a series of growth units extending in a uniform direction from a single (monopodial) or several (sympodial) meristems (Room et al., 1994), and “branching system,” which refers to the arrangement of branches inside an individual plant (Edelin, 1991). Depending on the objective of developmental processes, various formalisms have been proposed, of which the most commonly used language-based approaches, using L-system (Lindenmayer System) grammar, are one (Prusinkiewicz et al., 1996).FSPMs such as L-peach (Allen et al., 2005), L-Kiwi (Perttunen et al., 1998; Cieslak et al., 2011a) and IMapple (Cieslak et al., 2009) are the most important FSPM models that help to explain the source-sink relationships. The “GreenLab model” is a classic stochastic, discrete-mathematics functional-structural plant model that combines functional and structural representations of physiological processes, with “phytomers” as the elemental repeats. FSPM takes into account other important components such as photosynthesis, respiration, nutrient uptake, light interception, canopy structure, architecture and root system.

### 6.2 Structure and architecture

The architectural models of trees identify the fundamental elements of tree growth and development (Hallé et al., 1978). The basic architectural forms and their repetitions are taken into consideration in these models. The structure of plants results from a series of elemental repetitions, including “metamer,” “phytomer,” “axis,” “branching process,” and “growth unit,” with these architectural shapes and their repetitions being a prominent focus of these models. The important examples of architectural models related are given in **Table 2**.

**Table 2:** List of models using different elemental repeats and their application on fruit crops.

Name of the model	Crop scientific name	Elemental repeats	Reference
LIGNUM	*Pinussylvestris*L.	Growth unit	(Perttunen et al., 1998)
INCA	*Juglans regia*	Growth unit	(Le Dizès et al., 1997)
L- KIWI	*Actinidia deliciosa*	metamer	(DeJong, 2022)

#### 6.2.1 Canopy architecture

The architecture description of the plant is mainly based on topology, composition and geometry (Godin, 2000; Godin and Sinoquet, 2005; Prusinkiewicz et al., 2007). Several models operating on canopy architecture, referred to as architectural/geometrical models, have been documented (Hallé et al., 1978).

#### 6.2.2 Composition

The makeup of a plant comprises many types of materials. The architecture of plants is effectively delineated from the simplest to the most complex using representations such as modular, globular, and multi-scale (Godin, 2000). Plants are regarded as a whole in spherical representations, with geometric objects such as ellipses and cylinders used to depict the trunk and canopy. A plant is characterised by identifying any of the recurring units that constitute it, namely, metamers, growth units, and axes. (Harper et al., 1986) In a modular approach, multi-scale representation is fundamentally based on objects depicted at each scale, which elucidates the intricacy of plant architecture (Mandelbrot, 1982). The multi-scale tree graph (MTG) results from many tree graphs, each representing a distinct scale (Godin and Caraglio, 1998).

#### 6.2.3 Geometry

The spatial distribution of leaves, as influenced by the arrangement of roots, light interception, and its ramifications, is useful for predicting nutrient and water uptake efficiency (Prasad et al., 2022). A detailed relationship between plants and their micro-environment is well described by the Geometrical representations (Chelle et al., 1997).

#### 6.2.4 Topology

Topology is the mathematical and logical study of how elements in a space are connected and related, emphasising the structural properties that remain unchanged under continuous deformations. It is actually described using specific formalisms, such as the Lindenmayer system. In L systems, a module is characterised as recurring plant units, metamer, apex and branch, following a specific set of rewriting rules. The plant architecture explains effectively most of the problems related to carbon partitioning (Godin, 2000). The topology-based pipe model theory is used to mimic the thickness of stimulated stems and roots, as well as xylem and phloem conduits (Cieslak et al., 2011b; Jofre-Čekalović et al., 2022). The Pipe model theory proposes that the total cross-sectional area of stems and branches at a given height is proportional to the total number of leaves above that height (Grisafi et al., 2022). These models facilitate the representation of complicated branching systems by connecting unit pipes that symbolise plant components. The allometric relationships (Casella and Sinoquet, 2003; Yang et al., 2021) and the plant topology can be very well evaluated with the help of magnetic or sonic digitisers (Le Dizès et al., 1997; Sonohat et al., 2006; Belhassine et al., 2020) and photographs (Kaminuma et al., 2004), respectively.

#### 6.2.5 Aerial parts

Studying the types of shoots, organ growth and morphology, types of branches, and tree shape is crucial; for this aerial analysis, a stochastic approach known as a “Markov process” is employed (Costes et al., 2006). This process is Markovian, or “memoryless”, meaning that the future of the process can be predicted based only on its present state (Rabiner, 1989). Empirical models on a local scale were developed and used to evaluate the branching pattern along the trunks of different apple varieties (COSTES, 2002). Markov models were successfully applied to predict and experimentally define a sequence of distinct homogeneous zones along the trunk. These Markov models were efficiently applied to evaluate the zones observed in peach tree shoots of different lengths (Fournier et al., 1998). The transitional probability between two zones can be effectively evaluated using Markov and semi-Markov models (Taylor and Karlin, 1998). Notable instances of effective implementations of Markov and semi-Markov models in fruit crops include GrapevineXL for grapevines (Zhu et al., 2018), L-ALMOND for almonds (Negrón et al., 2013; DeJong, 2022), L-KIWI for kiwis (Cieslak et al., 2007), L-PEACH for peaches (Allen et al., 2005), and MappleTin for apples (COSTES, 2002).

#### 6.2.6 Root system

Root apparatus modelling is essential for accurately depicting the functioning of the entire plant (Pagès et al., 2000; Ndour et al., 2017). Mapping root architecture presents greater challenges than mapping the canopy, owing to the subterranean root system, necessitating invasive methods for investigation (Danjon and Reubens, 2008). In the case of FSPMs, roots are inadequately represented because they are collectively treated as a single module. The root system of herbaceous crops has been extensively investigated; nonetheless, challenges remain regarding root mortality and the structural roots of perennial fruit crops (Grisafi et al., 2022). **Table 3** presents some commonly utilised root-apparatus models for fruit crops.

**Table 3:** Application of root apparatus modelling in fruit crops.

Crop	FSPM model name	Purpose	Reference
Walnut	INCA	Root system is very simply described by three compartments (taproot, coarse root and fine root)	(Le Dizès et al., 1997)
Walnut	SIMWAL (SIMulatedWALnut)	Root system divided into compartments	(Balandier et al., 2000)
Plum	Dynamic 3D	Dynamic 3D representation of plum root system architecture with two information levels: (i) type of root axes and (ii) set of basic processes such as axial and radial growth, ramification and reiteration, and decay.	(Vercambre et al., 2003)
Kiwi	L-KIWI	Fibrous roots are the only type of root considered in our model of root development.	(Vercambre et al., 2003)
Almond	drainage lysimeter model	Empirical investigations concerning the impact of nutrition	(Sperling et al., 2019)

#### 6.2.7 Light interception

The light-harvesting capacity of the tree canopy plays a pivotal role in improving fruit tree productivity. There are various strategies for modelling the light interception in trees. One of the most adopted and effective tools is the Monte Carlo ray tracing method (Cieslak et al., 2008) to describe the path and interaction of multiple photons with the leaf surfaces (Disney et al., 2000). Recently, a new model, QuasiMS (Cieslak et al., 2008), was developed from this method and first successfully applied to the model L-KIWI (Cieslak et al., 2009). Some models that deal with light interception in the tree canopy are presented in **Table 4** below.

**Table 4:** Different models concentrating on light interception within tree canopies, accompanied by methodologies.

FSPM model name	Purpose	Approach which it is based on	Reference
The nested radiosity model	Modelling the distribution of natural light	Turbid medium approach	(Chelle and Andrieu, 1998)
RATP	Models the distribution of radiation absorption, transpiration and photosynthesis within the canopy space.	Turbid medium approach	(Sinoquet et al., 2001)
Quali Tree model	Calculation of photosynthetically active radiation (PAR) with respect to a canopy comprised of geometric shapes	Attenuation based on BeerLambert‟s law	(Lescourret et al., 2011)

#### 6.2.8 Photosynthesis and respiration models

The assessment of photosynthesis, a carbon framework and energy source for diverse biological processes, is conducted by recording the leaf area under certain light conditions at different time intervals and aggregating the total for the day’s photoperiod. The assessment of canopy photosynthesis can be achieved with a succinct programming language known as L-systems. **Table 5** presents the significant models used to calculate canopy photosynthesis, along with their applications.

**Table 5:** Models used in calculation of canopy photosynthesis.

Name of the model/approach/strategy used	Purpose	References
Big leaf model	Used to estimate canopy photosynthesis Daily response of canopy to intercepted radiation Incident radiation and fractional interception based on Beer’s law Exposed leaf photosynthesis	(Charles-Edwards, 1982)
“L-Systems	Using the patterns of plant development it is able to calculate light interception and canopy photosynthesis and was first used to model a peach tree.	(Lindenmayer, 1975)
Farquhar-von Caemmerer-Berry (FvCB) model	Biochemical model of C3 plant photosynthesis utilising a light response curve	(Farquhar et al., 1980)
Coupled approach	It takes into account ambient and foliar characteristics, in addition to stomatal conductance (gs).	(Collatz et al., 1991)

#### 6.2.9 Carbon partitioning

Net photosynthesis equals gross photosynthesis minus plant respiration. During photosynthesis in mature leaves, photosynthates are transported through the phloem to sink organs such as fruits, shoots and roots. The “Quali tree model,” a carbon partitioning model, is efficiently applied in Prunus persica, using carbon balance calculations based on the cumulative contribution of photosynthesis to carbohydrate reserves. Carbohydrates are allocated to each organ to support maintenance respiration and the growth of new leaf shoots, and subsequently to organs where carbon supply is insufficient for maintenance respiration. Finally, the remaining carbon is allocated to plants’ growth requirements. **Table 6** shows models of fruit trees and the concept of carbohydrate distribution.

**Table 6:** List of models used in carbon partitioning and their associated principles.

Name of the model	Principle by which the carbon flow is based on	References
LIGNUM	The functional balance according to the pipe model hypothesis delineates the correlation between biomass and tree cross-sectional area.	(Perttunen et al., 1998)
INCA	Potential source: carbohydrates pool and sink demands	(Le Dizès et al., 1997)
L-PEACH	Potential growth	(Allen et al., 2005)
L-KIWI	Carbohydrate availability from sources	(Cieslak et al., 2009; De Melo-Abreu et al., 2015)
L-ALMOND	Proximity of sources to the drain inside a tree structure and the impedance between the source and sink	(DeJong et al., 2017)
L-PEACH	Carbon transport resistance allocation model (CTRAM)	(Allen et al., 2005)
L-system approach	Munch hypothesis Michaelis–Menten sources and sinks	(Prusinkiewicz et al., 2000)
SIMWAL	According to the proportionate model, photosynthates distributed to each sink corresponded to its need without surpassing it.	(Balandier et al., 2000)
CLIM5-Fruit Tree	Carbon dynamics both in photosynthetic growth and in tree recovery at the end of the dormant period	(Dombrowski et al., 2023)

### 6.3 Uptake of nutrients through roots and hydraulics

The distribution pattern and architecture of roots need to be investigated to develop a plant feeding model (Habib et al., 1991). The experiment demonstrated that carbon uptake is primarily dependent on light intensity, whereas nitrogen uptake depends on soil nitrogen concentration (Drouet and Pagès, 2007). Some functional-structural plant models and plant-based models that have been further integrated with nutrition modelling are shown for peach (Rufat and DeJong, 2001) and grapevines (Wermelinger et al., 1991; Prieto et al., 2012; Zhu et al., 2018). The classical ‘Pipe model theory’ (Grisafi et al., 2022) describes well the xylem circuit, where stems and branches are considered as a collection of pipe units, each supporting one leaf (Grisafi et al., 2022). Examples of models that use this theory include L-KIWI and FAO (Food and Agriculture Organization). (Sonohat et al., 2006; GP et al., 2024) and HYDRUS-2D (Jovanović et al., 2023).

#### 6.3.1 Phenology-based models

The response of Perennial fruit trees to environmental cues is different and occurs at different rates. Parameters such as water availability, temperature, and solar radiation are key environmental factors regulating plant phenology (Zhao et al., 2013). Fertilisation, irrigation, pesticide application, harvesting, and estimation of the phenological stages require proper planning (Mullins et al., 1992; Ortega-Farías et al., 2002; Zavalloni et al., 2006), and here is where phenology-based models come into play. Phenological models are well suited to achieving objectives such as irrigation scheduling, fertilisation, and harvesting, thereby increasing use efficiency and reducing production costs and environmental risks to a great extent (Chmielewski et al., 2004). In the 1950s, the concept of “growing Degree Days” was introduced to develop phenological models (Baggiolini, 1952). The chilling hours model was developed in the 1800s and is a very reliable method for estimating the number of low-temperature units required to break a plant’s dormancy (Łysiak and Szot, 2023).

Global warming causes elevated winter and spring temperatures, which accelerates the premature growth of Spring apple phenophases, resulting in the disruption of their endo- and ecodormancy and increased vulnerability to future frost (Liu and Sherif, 2019; Parker et al., 2021). Four phenological models were successfully used to quantify late-spring frosts for the entire phenophase of apple trees (Malus domestica Borkh., cv. Fuji) in spring, considering two frost indices, i.e., accumulated frost days and accumulated frost degree days (Tan et al., 2021). A cohort-population concept-based unique simulation model (SIMBA-POP) was successfully used to predict phenological patterns and harvest dynamics in banana cropping systems. The model was also calibrated and validated using field data from the French West Indies (Guadeloupe and Martinique) for Musa spp., AAA group, cv. Cavendish Grande Naine. This model correctly predicts the dynamics of banana harvesting, in particular the date and the number of harvested bunches. The enhanced WOFOST (World Food Studies Model) model facilitates the prediction of jujube (*Ziziphus jujuba*) orchard yields across different ages by utilising the total dry weight (TDW) of newly formed organs. These models indicate that orchard age is one of the most crucial factors affecting the precision of fruit tree output predictions (Xu et al., 2024).

#### 6.3.2 Epidemiological models of fungal diseases

Climate significantly influences plant diseases by altering the physiology and resilience of the host (de Wit et al., 1970), as well as the rates at which infections proliferate. Abiotic stressors, including heat and dryness, alter defence mechanisms, hence diminishing plant resistance and increasing susceptibility to infections (Desaint et al., 2021). Precipitation, an essential element, influences the physiological composition of plants and the viability of pathogens, consequently impacting the dynamics of plant diseases (Colhoun, 1973; Milici et al., 2020). The prediction of grapevine development and the spread of powdery mildew using a comprehensive deterministic simulation model has been studied, and it is demonstrated that air temperature, wind speed, and direction are the main environmental input parameters (Calonnec et al., 2008). Brown rot of stone fruit is a devastating disease caused by *Monilinia* spp. It is widespread in temperate zones and affects economically important species such as peach, plum, apricot, cherry and almond (Oliveira et al., 2016). The Susceptible Exposed Infectious (SEI) model was useful in analysing the temporal dynamics of brown rot in fruit orchards and assessing the marketable output (Stetter and Lieb, 2000). The compartmental epidemiological model for brown rot was coupled to the fruit tree growth model to evaluate the effect of agronomic techniques on fruit quality (Bevacqua et al., 2018).

For stone fruits, compartmental SIR-type (Susceptible-Infected-Removed) epidemiological models are more efficient, accurately mimicking varied epidemics and thoroughly analysing climate change impacts on disease-induced yield losses (Bevacqua et al., 2023). This intelligent climate-driven model can also model epidemic patterns, using temperature and precipitation as the main parameters, to elucidate the synergistic effects on pathogen susceptibility in peach trees. The agricultural pest forecasting software developed by the computer centre in the 1960s is effective in predicting 13 insect species, 2 illnesses, and 2 storage disorders. Technological developments and upgrades in CIPRA over the years have enabled the development of comprehensive epidemiological models that account for air temperature, relative humidity, and leaf wetness duration (Prasad and Mathyam Prabhakar, 2012).

#### 6.3.3 Insect control forecasting models

Alternative methods to reduce pesticide use and the implementation of integrated pest management are the primary focus of policymakers globally (Stetter and Lieb, 2000). The absence of location-specific models for predicting natural pest control, which rely solely on a generic model based on landscape configuration, significantly constrains the predictive management tool available to stakeholders (Alexandridis et al., 2021). A study utilising generic landscape models of natural pests and biocontrol agents in cherry trees indicates that conservation tillage and unique interactions amongst crop, pest, and biocontrol agents are limiting variables. Insect forecasting models fundamentally encompass the intrinsic traits of insects, including their developmental stages and environmental and host-related variables. Prasad and Mathyam Prabhakar (2012) reported on the SOPRA (Swiss Orchard Pest Forecasting System) forecasting model utilised in Swiss fruit orchards, focusing primarily on the optimisation of pest management monitoring systems (Samietz et al., 2007).

#### 6.3.4 Innovative machine learning models

To tackle numerous agricultural challenges and develop innovative solutions, it is imperative to integrate present environmental and precision agriculture research with ML approaches (Mohammed et al., 2022). Artificial neural networks are essential to machine learning and artificial intelligence, facilitating the development of numerous innovative models to address a range of emerging challenges. These models can highlight different practical challenges (Emamgholizadeh et al., 2015; Naroui Rad et al., 2015; Safa et al., 2018). Deep learning, an advanced domain of data science, is an extension of the study on artificial neural networks, encompassing convolutional neural networks (LeCun et al., 1989), recurrent neural networks (Schuster and Paliwal, 1997), and deep belief networks (Hinton et al., 2006). Convolutional neural networks (CNNs) have demonstrated superior performance compared to traditional image-processing techniques (Kaminuma et al., 2004). Among the several applications of CNN, the detection of fruit and flowers at different stages, fruit harvesting and grading, and the production of fresh fruit are the most significant variables associated with fruit production (Wang et al., 2022). The applications of CNN about fruit crops are presented in **Table 7** below. The amalgamation of machine learning, computer vision, and IoT applications would undoubtedly enhance the profitability of farmers and other sectors (Meshram et al., 2023). Machine learning models serve as an alternate technique to validate prediction models for date palm (Mohammed et al., 2023). Recent advancements include metabolomics-based machine learning models for enhancing fruit taste prediction (Fernie and Alseekh, 2022), identifying iron chlorosis in plants through deep learning (Majdalawieh et al., 2023), and predicting mango yield using machine learning techniques. Recently, Artificial neural networks integrated with IoT devices play a pivotal role in greenhouse climate management including automatic disease and pest identification (Lim et al., 2023).

**Table 7:** CNN models, their objectives, and the corresponding techniques.

Name of the CNN model	Objective	Technique which it is based on	References
Updated MobileNet model	Automatic disease in avocado	Image processing	(Thangaraj et al., 2020)
V2IncepNet	Identification of lesion regions on mango leaves, and diagnosis of anthracnose illness.	Pattern identification and image characterisation	(Venkatesh et al., 2020)
3-layer CNN	Automated classification and assessment of eight mango cultivars.	Image rotation, translation, zooming, shearing, and horizontal flipping	(Rizwan Iqbal and Hakim, 2022)
Yolo CNN	Automated detection of citrus huanglongbing	Deep learning methodologies and sensory detection	(Qiu et al., 2022)
Banana squeezeNet	Identification of pest and diseases.	Utilising deep learning and Bayesian optimisation	(Bhuiyan et al., 2023)
esDNN (efficient supervised learning-based Deep Neural Network)	Helps in the earlier automatic detection of mango leaf diseases	AI-driven solution for detection and classification	(Gautam et al., 2024)
Yolo papaya	Timely identification of diseases	YoloV7 detector incorporating a CBAM attention mechanism	(de Moraes et al., 2023)
Citrus	Estimating fruit production by counting them	YOLO V 8 and Faster R-CNN	(Pichhika et al., 2025)
WOFOST-N	Simulate pear tree growth and nitrogen dynamics	WOFOST model with nitrogen module	(Xu et al., 2024)
CSS_PEAR	Simulate growth, production and quality of pear	Leaf and crop area indices	(De Melo-Abreu et al., 2015)
AppleQSM	Quantitatively analyse architectural in HDP	Uses data from TLS Terrestrial Laser Scanning	(Qiu et al., 2024)
VCHERRY	Sweet cherry growth and cropping	Formation and compilation of individual meristems	(Calonnec et al., 2008)
Olive Can	Determination of growth an productivity	Water and carbon balances of the orchard	(López-Bernal et al., 2023)

### 6.4 Comparative analysis of crop modelling approaches

Crop modelling approaches vary in their underlying principles, data requirements, computational demands, and practical applications. Empirical models are relatively simple, require fewer input variables, and are computationally efficient, making them well suited for rapid yield prediction and regional-scale studies. However, because they are based on statistical relationships rather than biological processes, their predictive performance often declines when applied under environmental conditions different from those used during model development. In contrast, mechanistic (process-based) models simulate the physiological processes that regulate crop growth and development, providing greater biological insight and making them more suitable for assessing climate change impacts and evaluating crop management strategies. Their application, however, is often constrained by the need for extensive calibration, high-quality datasets, and greater computational resources.

Functional–Structural Plant Models (FSPMs) further extend process-based modelling by integrating three-dimensional plant architecture with physiological processes. This enables detailed simulation of canopy development, light interception, source–sink interactions, and carbon allocation, making FSPMs particularly valuable for perennial fruit crops. Despite these advantages, their complex parameterisation, high computational requirements, and dependence on detailed architectural data have largely limited their use to research applications. In comparison, AI and ML models are data-driven approaches that can learn complex nonlinear relationships from large datasets without explicitly representing crop physiology. These models have shown remarkable success in yield prediction, disease diagnosis, fruit quality assessment, and precision orchard management. Nevertheless, their performance depends heavily on the availability of large, high-quality labelled datasets, and their limited biological interpretability remains a major challenge.

Overall, empirical models are widely adopted because of their simplicity and ease of implementation, whereas mechanistic and FSPM approaches provide deeper biological understanding at the expense of greater computational complexity and data requirements. AI and ML models offer excellent predictive accuracy and scalability, particularly when integrated with remote sensing, IoT, and high-throughput phenotyping technologies, although their “black-box” nature can limit interpretability. Consequently, hybrid modelling frameworks that combine the biological realism of process-based models with the predictive capability of AI are increasingly recognised as a promising direction for developing robust, interpretable, and scalable decision-support systems for sustainable fruit crop production (**Table 8**).

**Table 8:** Comparison of fruit crop modelling approaches.

Feature	Empirical models	Mechanistic/process-based models	FSPMs	AI/machine learning	References
Biological interpretation	Low	High	Very High	Low	(Grisafi et al., 2022)
Prediction accuracy	Moderate	High	High	Very High (with sufficient training data)	(Ajith et al., 2025)
Data requirement	Low	High	Very High	Very High	(Grechi et al., 2025)
Computational requirement	Low	Moderate	Very High	High during training; Moderate during prediction	(Grisafi et al., 2022)
Scalability	High	Moderate	Low	Very High	(Ajith et al., 2025)
Climate change assessment	Limited	Excellent	Excellent	Moderate	(Wang et al., 2024)
Practical adoption	High	Moderate	Mainly research applications	Rapidly increasing in precision horticulture	
Major limitation	Poor extrapolation	Complex calibration	High computational demand	Black-box nature and large data requirement	(Ajith et al., 2025)

## 7 Advances in modelling for fruit crops

### 7.1 Almond

Research findings demonstrated that the two-source energy balance (TSEB) model was effectively utilised to analyse actual evapotranspiration in almond orchards under diverse irrigation regimes. To achieve precise and reliable tree-level yield estimation of almonds, a CNN model was utilised to analyse the multi-spectral reflectance imagery (Tang et al., 2023). Similarly, the soil water balance for each orchard including almond was estimated through computing the crop evapo-transpiration by using FAO56 dual-Kc technique and the SIMDualKc (Simulation Dual Crop Coefficient Model) model (Ramos et al., 2023).

### 7.2 Apple

To predict the thinner response of apple fruitlets, the fruitlet growth model was effectively employed (Greene et al., 2013). The model operates on the premise that a fruit will detach only if its growth rate is less than 50% of the fastest-growing fruit on the tree during the same growth period, and will remain attached if its growth rate exceeds 50% of that fastest-growing fruit’s rate. Apple plant diseases were effectively identified using a CNN from leaf photos, and this information was made publicly accessible for detecting scab, black rot, and cedar rust (Vishnoi et al., 2023). The STICS model was found to be more successful in simulating apple phenology, yield, and yield loss from frost damage (Vishnoi et al., 2023). A recent study indicated that the fruiting capacity of apple trees, determined through meticulous segmentation of the trunk and branches, was assessed using the YOLOv8 (You Only Look Once version 8) model (Ahmed et al., 2025).

### 7.3 Apricot

The reduction in fruit quality deterioration caused by mildew, browning, and sand dust was effectively assessed using mathematical models under low-temperature, low-humidity conditions. The most valid rationale for the mathematical model of apricot drying in a natural environment was presented (Hu et al., 2022). Numerical models were employed to forecast the flowering date of apricot trees, thereby augmenting the profitability of fruit production, utilising experimental data on plant emergence, temperature, and photoperiod (Korsakova et al., 2023). The Radial Basis Function (RBF) neural network model has yielded profound insights into the diameter and weight of apricots during the growing season, enhancing understanding of production quantity and quality (Jannatizadeh et al., 2023).

### 7.4 Banana

Phenological models were employed to study the long-term effects of banana, and the simulations showed that climate, planting date, and nematicide application are the crucial factors affecting the fosthiazate concentration in fruits. This helps farmers reduce the number of harvested bunches with fosthiazate residues exceeding a threshold. For the prediction of the severity of Panama wilt disease and its impact on the arrangement of leaves, colour and shape, an agro deep learning model, a decision support system, was efficiently employed, which helps the farmers in making timely decisions (Sangeetha et al., 2023).

### 7.5 Cherry

According to the assessment of competition between tree crops and grass about transpiration, the CRPSM (Cherry Rootstock Permanent Sod Model, or defined according to the cited paper), a transpiration-yield model, effectively elucidates the variations in tree growth and productivity (Anderson et al., 1992). The management of Spotted Wing Drosophila, a significant pest, presents a considerable challenge due to its detrimental effect on Michigan’s tart cherry production. The Growing Degree Day-Based (GDD)-IPM model has been demonstrated to be the most effective in determining the optimal timing for pesticide application, utilising partial budget analysis, daily meteorological data, and phenological data (Durmaz, 2023). A method for predicting the canopy light distribution in cherry trees, utilising a three-dimensional (3D) point cloud model of the cherry tree canopy, was developed to facilitate informed tree pruning (Yin et al., 2023).

### 7.6 Citrus

To evaluate the response of citrus to various water-saving irrigation management strategies, FAO-56 (Food and Agriculture Organization Irrigation and Drainage Paper No. 56) agro-hydrological model was used efficiently in citrus orchards (Rallo et al., 2017). An integrated sugar-content prediction model, derived from a non-linear neural-network model, was utilised successfully in three species of citrus genus and this accurately measures the sugar content of the fruits in non-destructive manner and thereby enabling the famers to supply high quality, high value fruits (Kim et al., 2023).

### 7.7 Dragon fruit

The respiration kinetics of dragon fruit under different storage settings was effectively assessed using the Michaelis-Menten respiration model (Ho et al., 2020). The identification of dragon fruit mellowness and the determination of harvest time were effectively assessed using a deep learning convolutional neural network model, specifically RESNET (Vijayakumar and Vinothkanna, 2020; Kanso et al., 2023).

### 7.8 Grapes

A decision support tool, namely STICS (Simulateur multi disciplinary pour les Cultures Standard), was found to be effective for developing strategic planning in Portuguese viticulture. This model evaluates the effects of climate change on numerous related parameters and simulates water stress, yield, and phenological stages, thereby facilitating effective vineyard operations and winemaking methods for farmers (Fraga et al., 2020). It was utilised to assess the potential effects of heat waves on European wine-growing regions, facilitating the implementation of optimal mitigation strategies (Fraga et al., 2020). The AquaCrop model replicated fruit production, biomass, evapotranspiration, canopy cover, and soil-water content, facilitating irrigation scheduling and allowing farmers to evaluate water productivity in dry and semi-arid environments (Er-Raki et al., 2021). Catering to the need for higher-quality wine, accurate fruit counts are a crucial factor and therefore a necessary decision-making step before harvest. Currently, no such reliable counting techniques are available. The tracking and counting of grape clusters in the field, on a real-time scale, based on video data, was made possible with the introduction of a lightweight cluster detection model, YOLOv5, which works based on a channel pruning algorithm and SORT algorithm (Shen et al., 2023). Similarly, the grape disease identification in real-time, using an improved YOLOXS (You Only Look Once Extra Small) GFCD-YOLOXS (Grape Fruit Cluster Detection–YOLOXS), has been reported (Wang et al., 2023). There are two major components of this model, namely FOCUS and CBAM, which are used to enable faster, more efficient disease identification.

### 7.9 Mango

The functional-structural plant model, V-Mango, employs a multi-scale method to simulate the cultivation characteristics of mango by modelling its intricate architectural evolution across several growing seasons (Grechi et al., 2025). A study utilised a unique mathematical model that combined an enzyme kinetics-based respiration rate model with the Arrhenius equation to develop an active modified atmospheric storage system for 100 kg of mango, maintained at a temperature of 27 °C (Thakur and Mangaraj, 2022). In forecasting mango production, the existing ARIMA (AutoRegressive Integrated Moving Average) model, along with artificial intelligence techniques such as Neural Network Autoregressive and non-linear support vector regression models, has demonstrated greater efficacy (Rathod and Mishra, 2018).

### 7.10 Mangosteen

For understanding the growth and form complexity of Mangosteen, the role of mathematical models using fractals and computers is highly appreciable (Puzon, 2005). A deep convolutional neural network model, namely, ResNet50, was much effective in classifying the ripeness of mangosteen and enabling to classify according to the market segments (Arampongsanuwat and Chaowalit, 2021).

### 7.11 Olive

In a study conducted in olive orchards in Spain, the stochastic weather generator ClimaSG was found to be the best for estimating crop water requirements and irrigation under rainfed and irrigated conditions (Mairech et al., 2022). In China, the successful use of a deep learning model, U2, to estimate growth and fruit yield helped farmers manage olive orchards (Ye et al., 2022). For analysing the effect of cover crops on minimising erosion rates, the process-based simulation model Olive can was found to be best suited to olive orchards (López-Bernal et al., 2023).

### 7.12 Papaya

The growth dynamics of papaya fruit and its thermal requirements, quantified as GDD from bloom to ripening, are crucial for predicting fruit size and harvest dates. Research conducted in this area demonstrated that the employed model effectively predicted fruit growth and the GDD needed for papaya ripening (Salinas et al., 2019). With its simplicity and excellent fitting results, Gompertz equation successfully described papaya fruit growth which followed a straightforward sigmoid curve.

### 7.13 Passion fruit

A crop-specific and dynamic pesticide uptake model, termed DynamiCROP, useful for advising farmers on the judicious choice of pesticides and their application in passion fruit. This model takes into consideration of the inputs like amount of spray deposition of various pesticides on plant surfaces, its uptake mechanisms, dilution, degradation, etc (Juraske et al., 2012).

### 7.14 Peach

For simulating the impact of change in climate on brown rot disease and epidemic patterns in peach orchards, a compartmental SIR- type epidemiological model has been utilised (Bevacqua et al., 2023). And it is evident from the model that the temperature and precipitation are the main causal agents for brown rot epidemics in peach. It was also observed that there is a synergy between alterations in crop phenology and vulnerability to pathogens. A kinetic model, namely Ordinary differential equations kinetic model, was found to the best in simulating the various developmental stages along with sugar accumulation in peach (Kanso et al., 2023). Using linear mixed-effects models, a study was conducted to investigate the effect of rootstock micropropagation on peach varieties grafted with rootstocks propagated, with the main objective of identifying the impact of environmental and cultural factors on the yield in the woody plants (Marín et al., 2023).

An accurate prediction on internal fruit quality in terms of dry matter content and maturity of seven peach cultivars was made possible by integrating visible-NIRS (near-infrared spectroscopy) with individual cultivar-specific data (Anthony et al., 2023).

### 7.15 Pecan nut

For predicting different nut growth stages in pecan, embryo growth models utilise phenotypic traits. From different non-linear growth models reported, it was observed that Gompertz model and the logistic model, fit best to describe embryo development and modelling shell growth, respectively (Panta et al., 2023). These models play a pivotal role in the analysis of irrigation scheduling in pecan and reveal that thinning at the late stages before nut filling and the minimum use of pesticides at the shell lignification stage will help maximise productivity.

### 7.16 Pineapple

ALOHA PINEAPPLE v. 2.1, a process-oriented model, used to simulates the growth, development, and production of the mother plant of the pineapple (variety - Smooth Cayenne). This model operates on a daily time step and takes into account inputs such as daily meteorological data, soil profile characteristics, and management information (Zhang et al., 1997). Another classical model, namely the “SIMPIÑA model”, simulates the growth and development of the “Queen Victoria” pineapple cultivar in response to stress induced by nitrogen and water deficiency. AlexNet deep learning models are successfully used in Thailand to categorise sweetness levels based on physical attributes, as the market price of pineapple is determined by its sweetness (Kanso et al., 2023).

### 7.17 Strawberry

By evaluating some parameters of strawberries (L, b, TSS, and firmness) at different ripening stages using combinations of spectral reflectance indices (SRI), integrated with ML models, including artificial neural networks (ANN), random forests (RF), and decision trees (DT), the optimal harvest time of strawberries can be efficiently determined (Elsayed et al., 2025).

### 7.18 Pear

Modifying the WOFOST model called Python Crop Simulation Environment (PCSE) can be an efficient way to estimate the effect of soil, weather conditions, pruning and irrigation on the growth of fruit trees and water transport in the orchards of Pear (Wang and Song, 2022).Assimilating the LAI and soil moisture into the WOFOST Model Based on Satellite Remote Sensing Data increased the accuracy of pear yield prediction (Fan et al., 2025).

## 8 Bottlenecks in the application of crop modelling

### 8.1 Input data

Complete and accurate experiment data are needed to run a model. Inaccurate or missing data can lead to false conclusions. For accurate findings, a year of data input is necessary, particularly in perennial fruit crops. The model must have the provisions to accommodate the variability; otherwise, it will affect the output, independent of data quality (Paeth et al., 2008).

### 8.2 Knowledge

To utilise the model, the user needs computer skills as well as agricultural knowledge relevant to the experiment. Additionally, every model functions differently. Therefore, understanding the particular software to be used is crucial (Pagès et al., 2000).

### 8.3 Complexity of biological systems

Generalised models do not account for the complex nature of a particular crop. Moreover, many fruit crops have complex flowering and bearing habits, making them difficult for the software to understand. Reliable projection of changes under climate variability on a local scale, such as storms and droughts (Pagès et al., 2000) is yet another bottleneck for models.

### 8.4 Models don’t eliminate the need for continued experimentation

Although virtual experiments make the work easier, models cannot be run without practical information about the crop. Due to limited available knowledge and the experimental difficulties associated with roots, which do not evolve through the generation of metamers, researchers still have difficulty simulating root architecture (Pagès et al., 2000).

### 8.5 Region-specific

The calibration procedure is challenging because most models are developed based on the information and experiments done in a particular location. However, it may also be noted that a crop’s requirements differ depending on where it is grown. For example, cherry trees are primarily used as ornaments in Japan.

## 9 Challenges and future line of work

Crop models are useful tools to perform agricultural research and support decision making, but their widespread use is still constrained by a number of practical issues. Many models require detailed, high quality data on weather, soil, crop management and cultivar characteristics which are frequently absent or inconsistent across regions. The calibration, validation and operation of these systems require specialised technical expertise. This limits their use by farmers and extension personnel. Moreover, the lack of digital infrastructure, limited access to standardised datasets and poor interoperability between modelling platforms, as well as insufficient training and extension support, hinders large-scale implementation. Overcoming these challenges through standardised databases, user-friendly decision support systems, improved data sharing frameworks and capacity building will be critical for enhancing practical adoption of crop modelling for sustainable agriculture.

Although crop models are basically helpful for researchers and policymakers, they are not considered handy for farmers due to the lack of the technical knowledge required to run them, they can still serve the farming community well. Farmers, in collaboration with experts, can seek information and guidance on their crops and crop plans to minimise the risk of crop failure. Moreover, crop models are available as mobile applications. With the recent advancement in the field of genetics and molecular plant biology, improving crop responses of crop modelling to environmental conditions and management factors, is a new area to explore in the future (Shafique et al., 2007). In Philippines, a rice crop management model, Rice Crop Management (RCM), was released as a mobile application so that as many farmers as possible could benefit from its simplicity. Similarly, in Nebraska, an irrigation-based model application called Corn-Soy Water was released to benefit the farming community. However, the use of this application is limited to the country of release itself. The key problem that hasn’t been solved is that modellers are usually accused of taking credit from the data provider. Enhancing current models and other decision aids is constantly a work in progress and will always be available. To create a worldwide programme with a minimum data set (MDS) for soil, crop, and meteorological variables, we must gather and organise all currently available data using a geographic information system (GIS) to develop a user-oriented decision support system. Because new decision aids are being developed so quickly, it is essential to train a critical mass of researchers, educators, administrators, and policymakers to maximise potential benefits.

Future fruit crop modelling research should prioritise the development of hybrid modelling frameworks that integrate mechanistic crop models with AI, ML, and deep learning techniques. Such approaches can combine the biological interpretability of process-based models with the predictive power of data-driven methods, enabling more accurate simulation of complex physiological processes under variable environmental conditions. Another important direction is the development of digital twins for fruit orchards, which integrate real-time data from IoT sensors, UAVs, satellite remote sensing, weather stations, and crop models to support dynamic decision-making for irrigation, fertilisation, pest management, pruning, and harvest scheduling. In addition, future models should improve the representation of below-ground processes, including root architecture, water and nutrient uptake, rhizosphere interactions, and rootstock–scion relationships, whilst incorporating genomic, phenomic, metabolomic, and microbiome datasets to enable genotype-specific simulations and accelerate the development of climate-resilient fruit cultivars.

Future research should also focus on developing standardised, open-access databases containing long-term weather, soil, crop management, and experimental datasets to improve model calibration, validation, uncertainty analysis, and transferability across regions. Incorporating ensemble modelling, probabilistic forecasting, and multiple climate change scenarios will enhance the reliability of predictions under increasing climatic uncertainty. Furthermore, integrating high-resolution remote sensing technologies, such as hyperspectral imaging, LiDAR, thermal sensors, and UAV-based phenotyping, will improve the monitoring of canopy structure, crop water status, nutrient deficiencies, disease incidence, and fruit yield. Finally, the development of cloud-based platforms, mobile applications, multilingual interfaces, and user-friendly decision-support systems will facilitate the wider adoption of advanced fruit crop modelling technologies by researchers, extension agencies, policymakers, and growers, thereby promoting precision horticulture and sustainable fruit production.

## 10 Real-world adoption of crop modelling technologies

Although crop modelling has advanced considerably over the past few decades, its adoption by fruit growers is still limited, especially in perennial orchard systems. Most modelling tools are currently used by researchers, academic institutions, and government agencies for research, climate impact assessment, and policy planning rather than for routine farm management. However, several crop model-based decision-support systems have been successfully implemented for applications such as irrigation scheduling, pest and disease forecasting, nutrient management, and harvest planning. These tools have demonstrated their ability to improve input-use efficiency, reduce production costs, and support timely management decisions in commercial fruit production.

The wider adoption of crop modelling depends on both technical and practical considerations. Many models require detailed information on weather, soil, crop, and management practices, as well as technical expertise for calibration and interpretation. These requirements can limit their use by growers, particularly smallholder farmers. In developing countries, adoption is further constrained by limited digital infrastructure, poor access to reliable datasets, the high cost of sensors and precision agriculture technologies, and inadequate extension services. In addition, the economic benefits of adopting crop modelling tools are not always immediately evident, making growers hesitant to invest in these technologies.

Expanding the practical use of crop models will require the development of simple, user-friendly decision-support systems that can integrate weather information, remote sensing, mobile applications, cloud computing, and IoT technologies. Improving extension services, increasing access to open databases, lowering implementation costs, and providing training for farmers and agricultural advisors will also encourage wider adoption. As digital agriculture continues to evolve, affordable and easy-to-use crop modelling tools are expected to become increasingly important for supporting precision horticulture and sustainable fruit production.

## 11 Conclusion

Models are comprehensive, knowledge-based tools with both local and international uses. It aids the assimilation of experimentally acquired knowledge and the understanding of the behaviour of biological systems at the underlying level of integration. Crop models are dynamic, quantitative tools that help analyse the agricultural system’s complexity. It enhances interdisciplinary research and maximises efficiency in agricultural research and management. The integration of main fundamental physiological processes with branching processes, is the main advantage of functional structural plant models.

However, research in the area of root architecture needs further attention due to the complexity of roots, as the development of roots is not represented by elemental repeats. Phenology-based models, though robust in simulating the growth and development of crops, efficiently help with the harvesting dynamics of horticultural produce. Existing epidemiological models well address the temporal outbreak of diseases in response to environmental factors. The added advantage of using a compartmental epidemiological model in fruit crops is the integration of management practices with fruit quality parameters. Insect forecasting models should progress in a direction where a shift from generic to specific levels is needed, with the inclusion of beneficial insects in a specific landscape, so that the decision-making process (use of plant protectants) of all stakeholders will become more efficient. The rise of novel machine learning models helps to address various issues in the popularisation of precision agriculture in developing countries in near future.
